# Supplementary material for: Characterization of a Hg2+-Selective Fluorescent Probe Based on Rhodamine B and Its Imaging in Living Cells
Source: Molecules. 2021 Jun 3;26(11):3385. doi: 10.3390/molecules26113385 (PMC8199853; doi:10.3390/molecules26113385)
Supplement: Supplementary file 1 [file molecules-26-03385-s001.zip › molecules-1186992-supplementary.pdf]

Characterization of a Hg^{2+}-Selective Fluorescent Probe Based on Rhodamine B and Its Imaging in Living Cells

Wenting Zhang [1,2†], Chunwei Yu [1,†], Mei Yang [2], Shaobai Wen [1] and Jun Zhang [1,3]**

[1] Laboratory of Environmental Monitoring, School of Tropical and Laboratory Medicine, Hainan Medical University, Haikou 571199, China; hy0211049@hainmc.edu.cn (C.Y.); wenshaobai@163.com (S.W.)

[2] School of Public Health, Hainan Medical University, Haikou 571101, China; zhangwt2021@163.com (W.Z.); myang_1995@163.com (M.Y.)

[3] Laboratory of Tropical Biomedicine and Biotechnology, Hainan Medical University, Haikou 571101, China; jzhang@hainmc.edu.cn (J. Z.)

* Correspondence: jzhang@hainmc.edu.cn; Tel.: +86-898-66973160

† These authors contributed equally to this work.

Contents

Figure S1 Influences of time on the fluorescence spectra of **P** (5 μM) with Hg^{2+} (50 μM) in ethanol solution ……2

Figure S2 Fluorescence response of 5 μM of **P** to 10 μM of Hg^{2+} and to the mixture of 10 μM of individual other metal ions a) and anions b) with 10 μM of Hg^{2+}……3

Figure S3 The fluorescence intensity at 583 nm of **P** (5 μM) as a function of Hg^{2+} concentrations (1-20 μM)……4

Figure S4 Benesi-Hildebrand plot of **P**, assuming 1:1 stoichiometry for association between **P** and Hg^{2+}……5

Figure S5 Reversible titration response of **P** to Hg^{2+}……6

Figure S6 ^{1}H-NMR spectrum of **P**……7

Figure S7 ^{1}H-NMR titration experiment of **P**+Hg^{2+}……8

Figure S8 MTT assay of **P** in living cells……9

Figure S9 ESI (-)-MS of **P**……10

Figure S10 ESI (+)-MS of **P**……11

Figure S11 ^{13}C-NMR spectrum of **P**……12

Figure S1

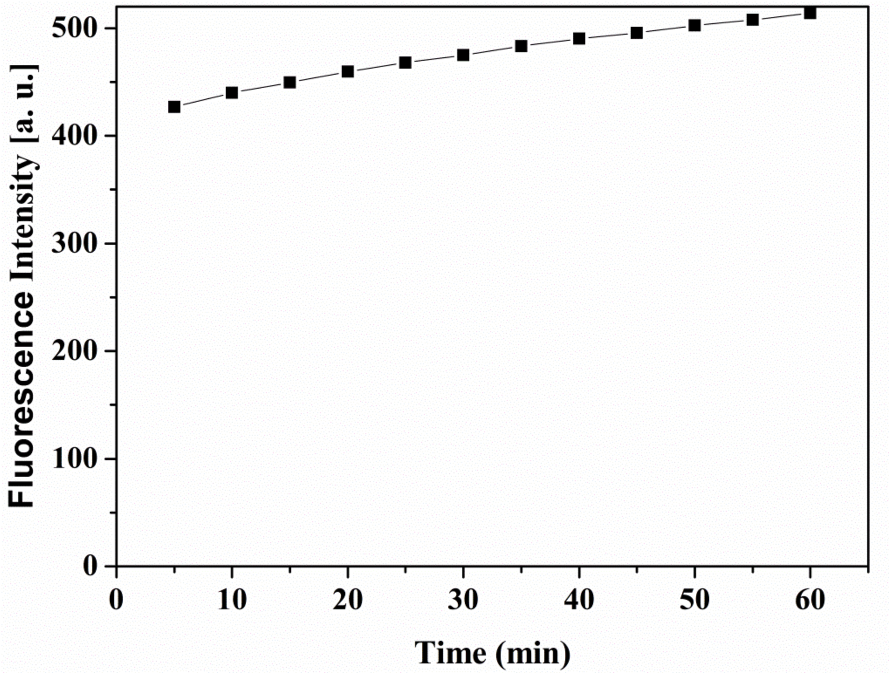

Figure S1 Influences of time on the fluorescence spectra of **P** (5 μM) with Hg^{2+}(50 μM) in ethanol solution.

Figure S2

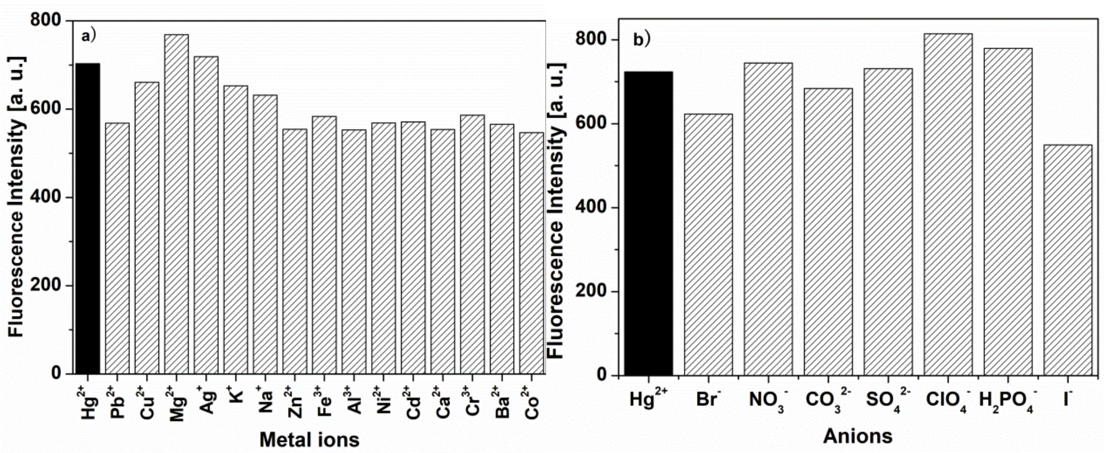

Figure S2 Fluorescence response of 5 μM of **P** to 10 μM of Hg^{2+} and to the mixture of 10 μM of individual other metal ions a) and anions b) with 10 μM of Hg^{2+}.

Figure S3

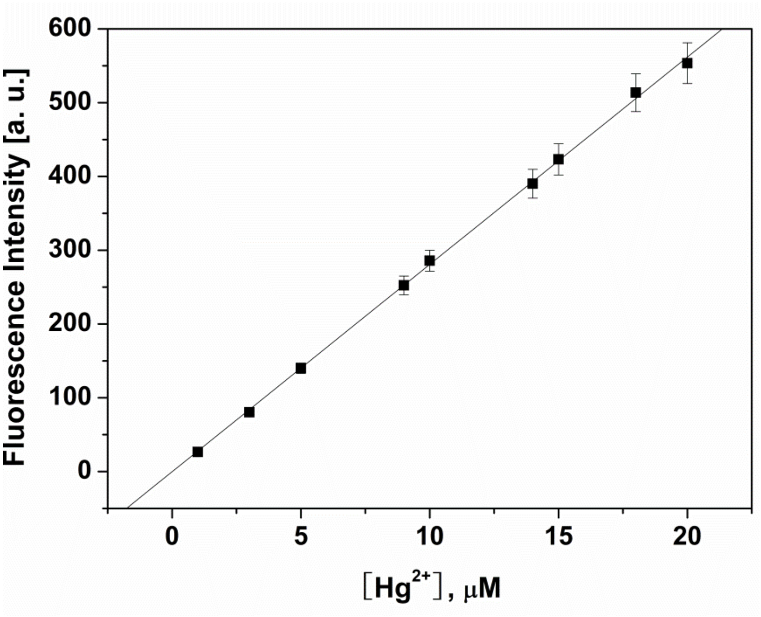

Figure S3 The fluorescence intensity at 583 nm of **P** (5 μM) as a function of Hg^{2+} concentrations (1-20 μM).

Figure S4

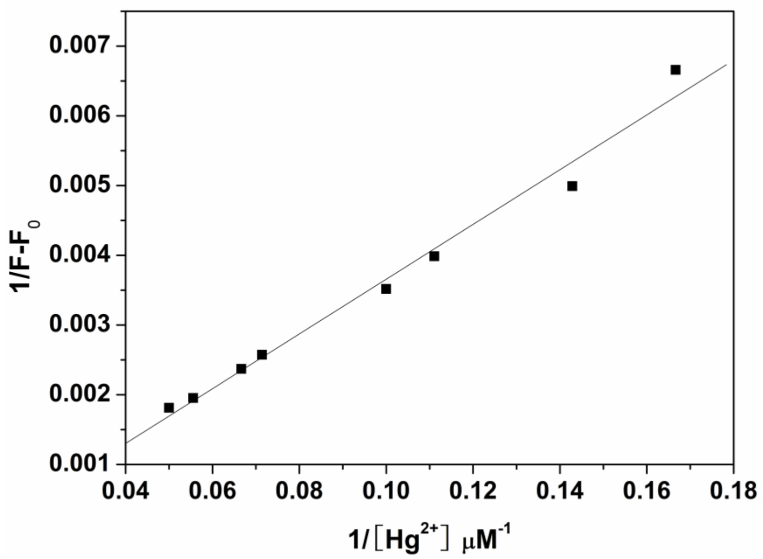

Figure S4 Benesi-Hildebrand plot of **P**, assuming 1:1 stoichiometry for association between **P** and Hg^{2+}.

Figure S5

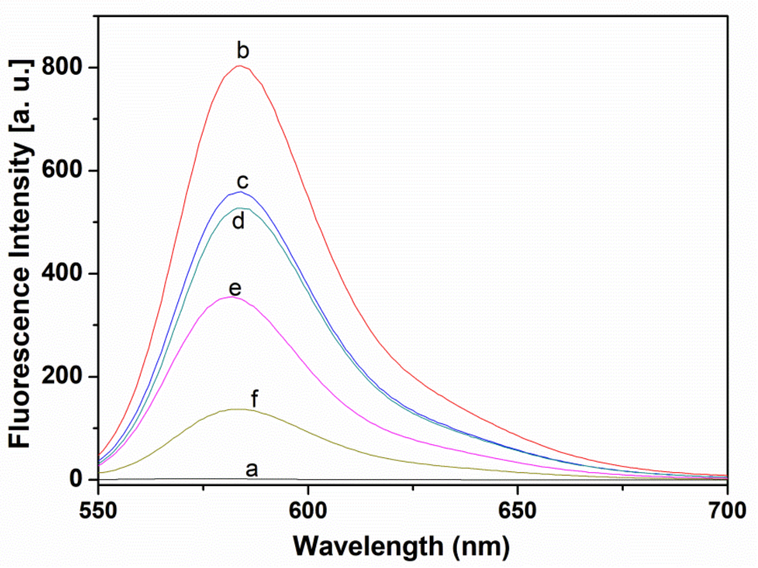

Figure S5 Reversible titration response of **P** to Hg^{2+} in ethanol-water solution (8:2, v:v, pH7.0, 20 mM HEPES). a: **P** (5 μM)**;** b: P (5 μM) with Hg^{2+} (50 μM); c: **P** (5 μM) with Hg^{2+} (50 μM) and then adding Na_2S (50 μM); d: **P** (5 μM) with Hg^{2+} (50 μM) and Na_2S (500 μM); e: **P** (5 μM) with Hg^{2+} (50 μM) and Na_2S (50 μM) and then addition of Hg^{2+} (500 μM); f: **P** (5 μM) with Hg^{2+} (50 μM) and Na_2S (500 μM) and then addition of Hg^{2+} (500 μM).

Figure S6

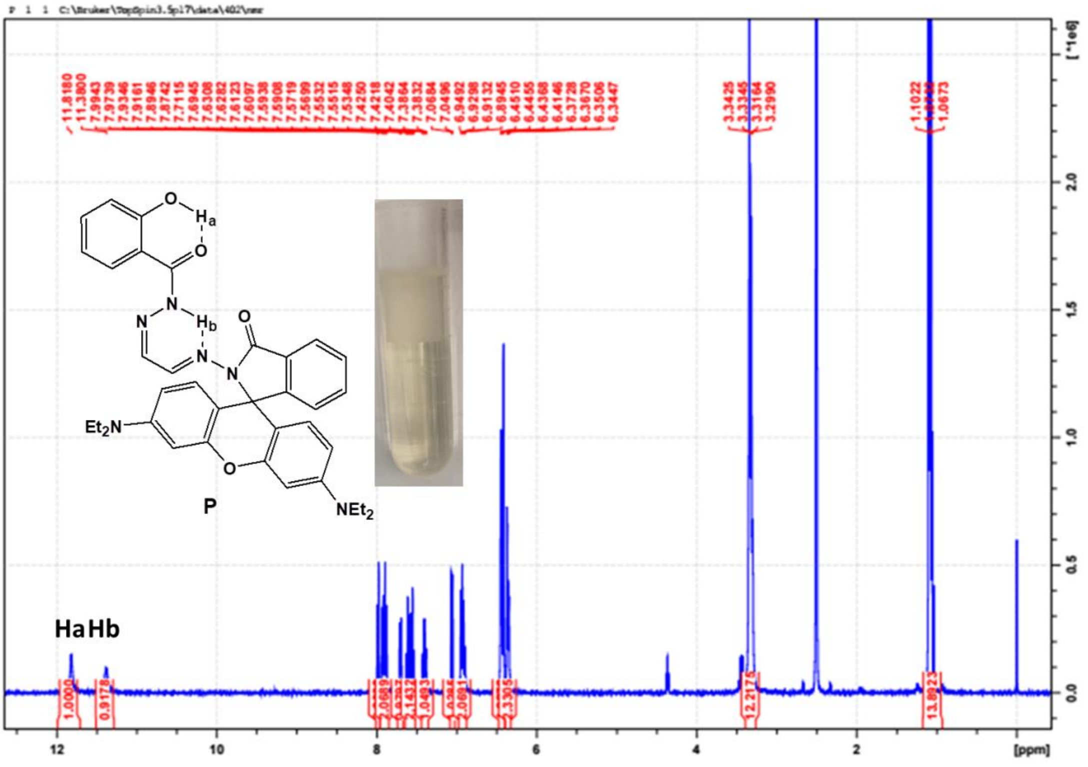

Figure S6 ^{1}H-NMR spectrum of **P**.

Figure S7

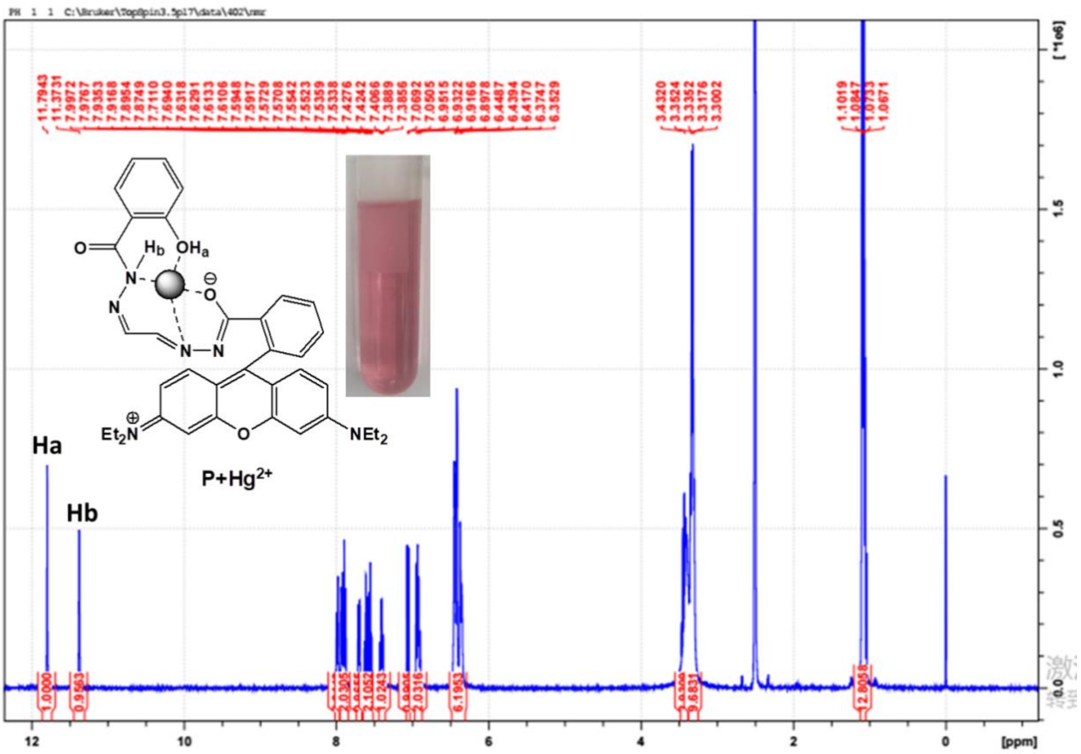

Figure S7 ^{1}H-NMR titration experiment of **P**+Hg^{2+}.

Figure S8

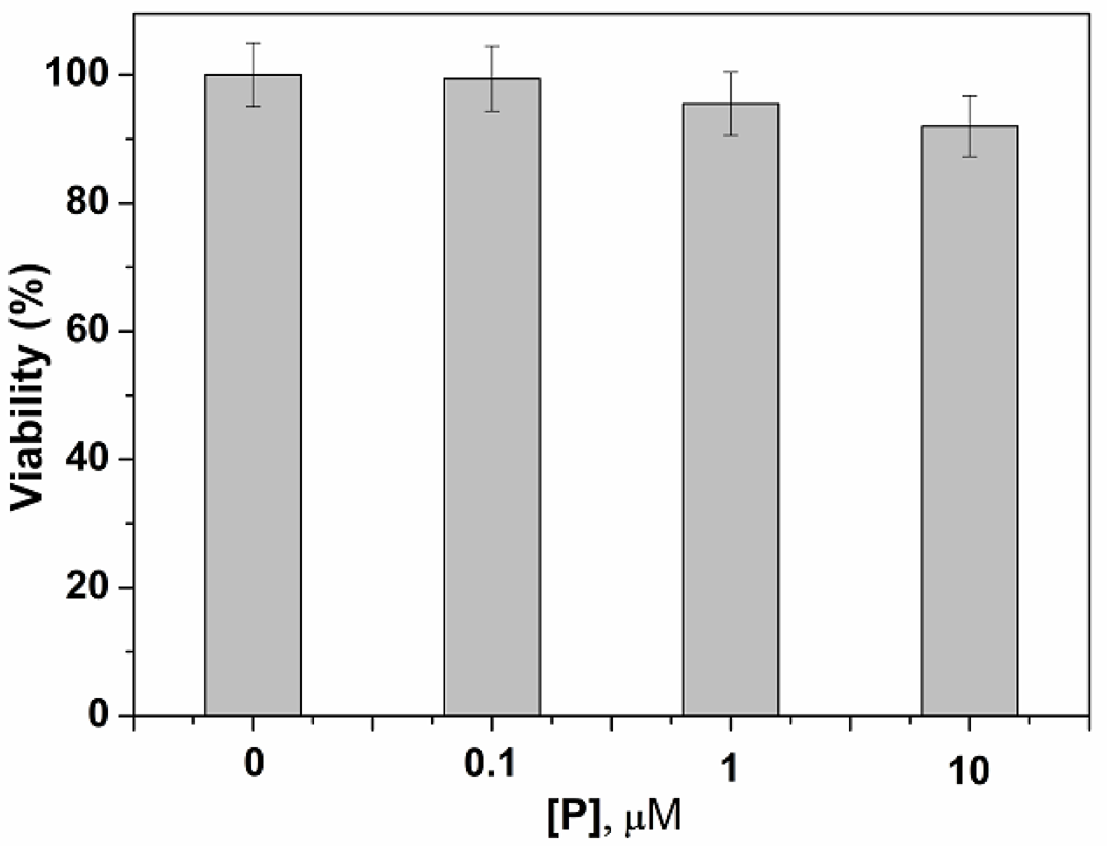

Figure S8 Cell viability values (%) estimated by MTT proliferation test versus incubation concentrations of **P**. PC12 cells were cultured in the presence of 0−10 μM **P**.

Figure S9

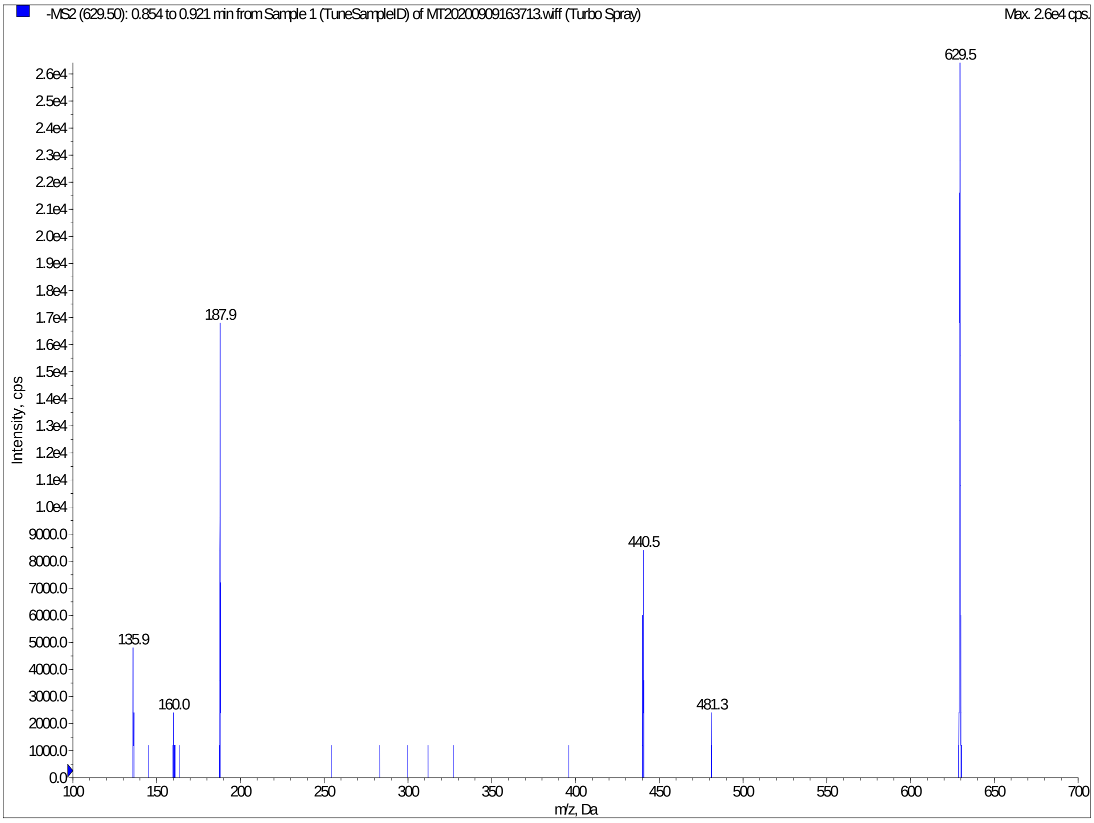

Figure S9 ESI (-)-MS of **P**.

Figure S10

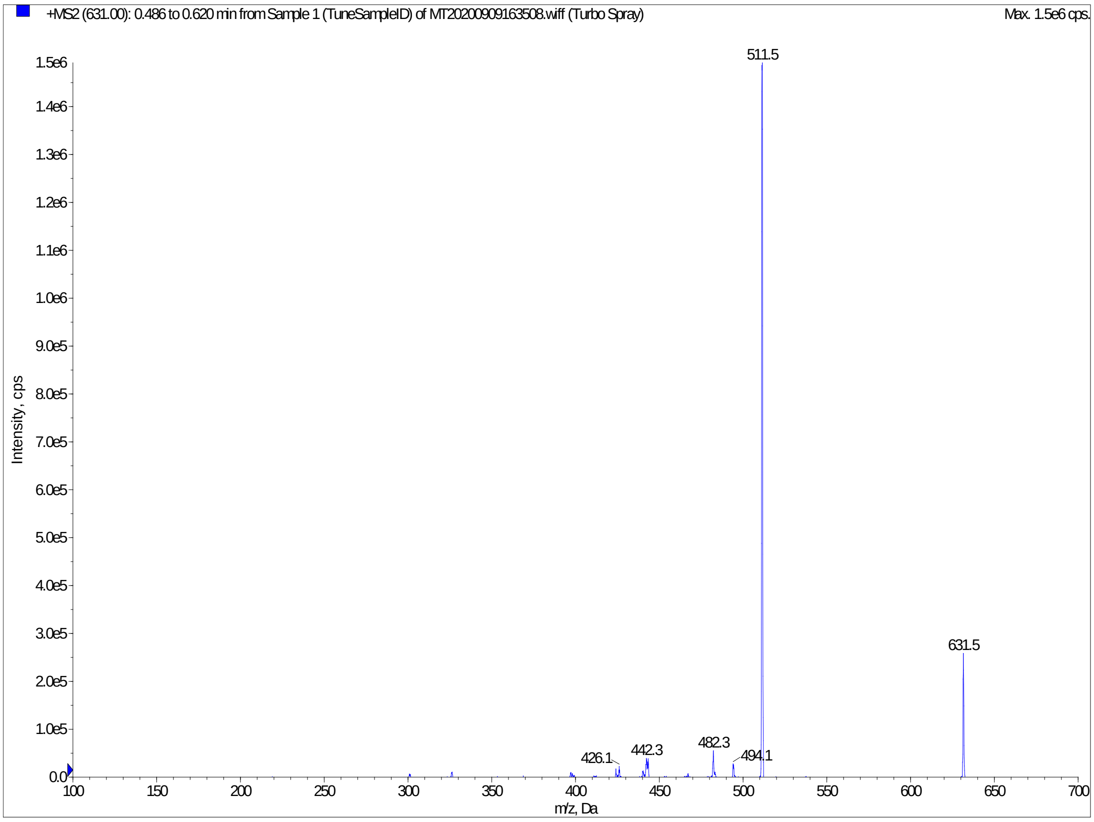

Figure S10 ESI (+)-MS of **P**.

Figure S11

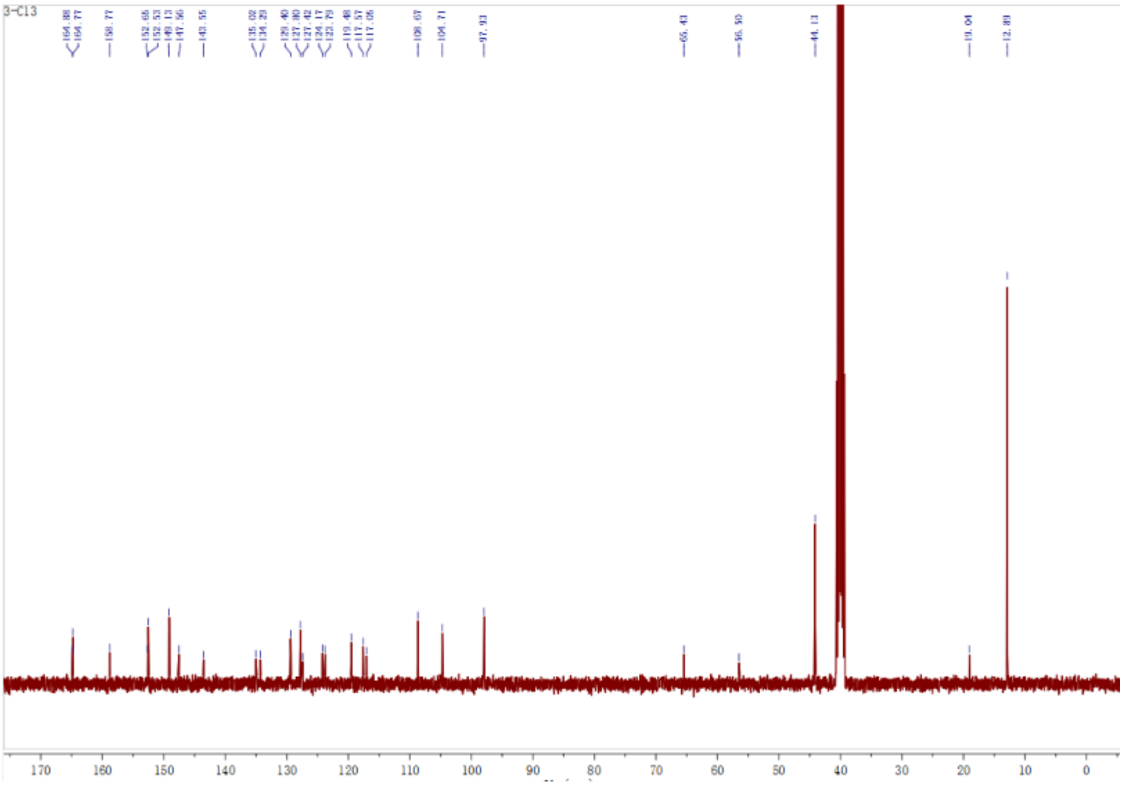

Figure S11 ^{13}C NMR spectrum of **P**.
